# Voriconazole-induced periostitis post lung transplantation

**DOI:** 10.1016/j.radcr.2022.01.078

**Published:** 2022-03-11

**Authors:** Orla M. Murray, John P Hynes, Michelle A Murray, Eoin C Kavanagh

**Affiliations:** a Department of Radiology, Mater Misericordiae University Hospital, Eccles Street, Dublin 7, Ireland; b Graduate Entry Medical School, University of Limerick, Limerick, Ireland; c Department of Respiratory Medicine, Mater Misericordiae University Hospital, Eccles Street, Dublin 7, Ireland

**Keywords:** Voriconazole, Periostitis, Lung transplantation

## Abstract

Voriconazole is a broad-spectrum triazole antifungal used to treat invasive fungal infections. It is commonly used prophylactically in immunocompromized patient cohorts, including transplant recipients. Diffuse periostitis is a very rare complication of chronic voriconazole use. It is associated with diffuse bone pain, elevated serum alkaline phosphatase and fluorine levels. Characteristic imaging findings include periosteal thickening with a dense, nodular, irregular and often bilateral pattern. We describe the case of a 71-year-old female who presented with multifocal bone pain six years following double lung transplantation. Her post transplantation course had been complicated by a life threatening episode of sepsis secondary to Scedosporium apiospermum, a rare invasive fungal infection following which lifelong prophylaxis with oral Voriconazole was commenced. We discuss the characteristic clinical and imaging manifestations of this rare condition.

## Case Report

A 71-year-old female presented with diffuse bone pain involving the shoulders and multiple ribs. On examination there was slightly reduced strength in both upper extremities, full although painful range of motion in the shoulder joints and diffuse tenderness at both shoulders and over multiple left-sided ribs. Her history was significant for double lung transplantation for emphysema six years previously. The post-transplantation course was complicated by severe sepsis due to Scedosporium apiospermum, a rare invasive fungal infection, requiring critical care admission. The patient was subsequently commenced on oral voriconazole as prophylaxis to mitigate against further fungal infection.

**Fig 1:**
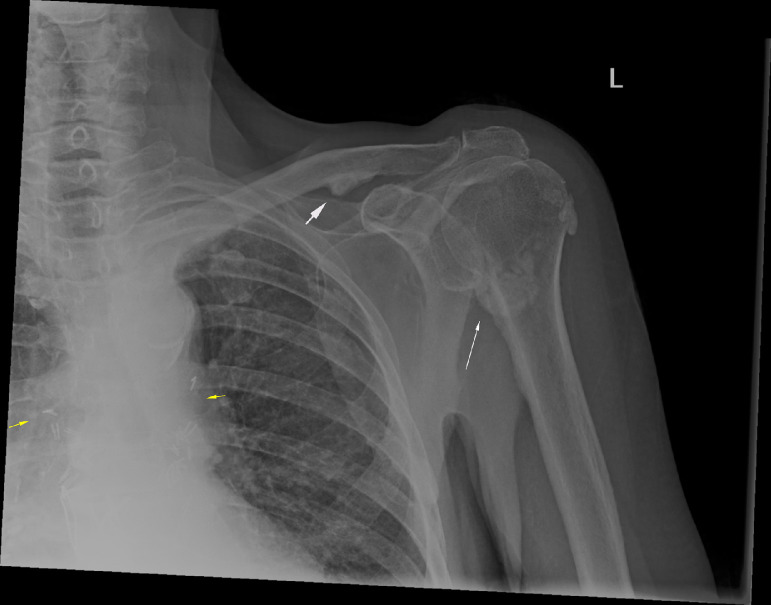
AP radiograph of the left shoulder: There are foci of juxta cortical mineralisation at the proximal left humerus (white arrow) and inferior left clavicle (white arrowhead) consistent with periostitis. Surgical clips are noted at the hila bilaterally (yellow arrows) in keeping with the history of bilateral lung transplantation.

**Fig 2:**
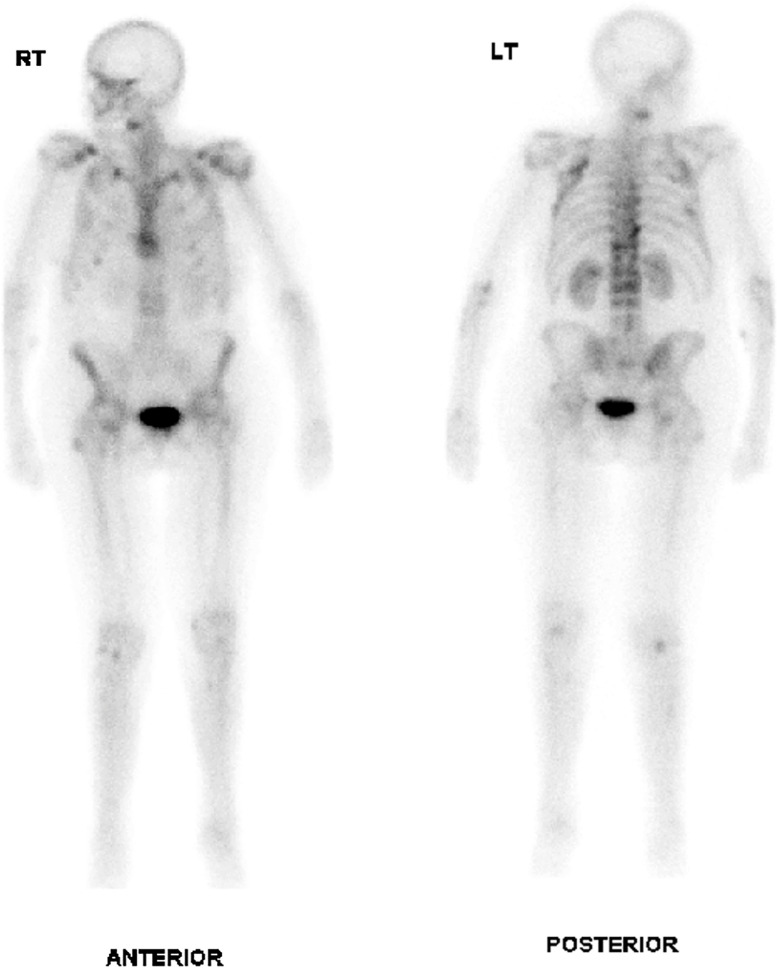
a and b. Whole-body radionuclide bone scan demonstrates multifocal radiotracer uptake in the ribs and left shoulder.

**Fig 3:**
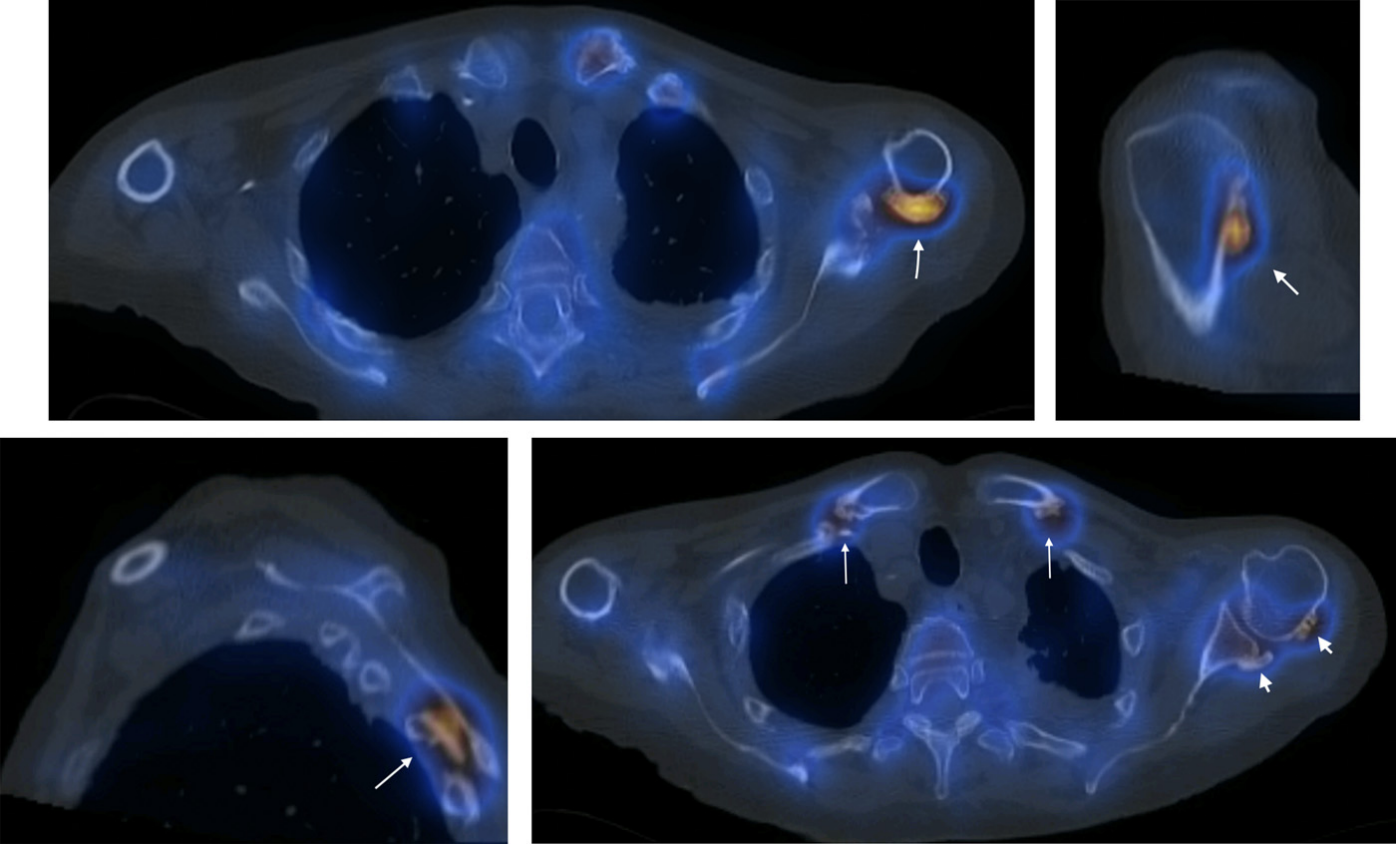
(a). Axial SPECT scan of the thorax demonstrates juxtacortical ossification at the posterior aspect of the proximal left humerus, associated with prominent tracer uptake (white arrow). (b). Parasagittal SPECT image of the left shoulder demonstrates juxtacortical ossification at the posterior aspect of the proximal left humerus, associated with prominent tracer uptake (white arrow). (c). SPECT image at the left parasagittal level shows juxtacortical ossification with tracer uptake in a left posterior rib (white arrow). (d). Axial SPECT image of the upper thorax demonstrates juxtacortical ossification bilaterally at the clavicles (white arrows) and at the left posterior humoral head and left glenoid with low grade increased tracer activity (arrowheads).

Radiographs of the chest and shoulders were obtained (Fig. 1). Changes of sclerosis and juxtacortical mineralization at the left proximal humerus and clavicle were noted. These findings were new in comparison to the patient's previous post-transplant imaging. Whole-body radionuclide bone scan (Fig. 2 a and b) and subsequently SPECT CT was performed to further characterize these appearances and demonstrated periostitis involving the clavicles bilaterally, both humeral heads worse on the left, left scapula and left third, fourth, and fifth ribs with abnormal tracer accumulation confined to the periosteum at sites of involvement (Fig. 3a, b, c, d**).** A diagnosis of voriconazole-induced periostitis was made based on these characteristic imaging appearances, in combination with the patient's history. This was supported by a significantly elevated serum alkaline phosphatase value of 220 U/L (normal range 30-130 U/L), a characteristic laboratory finding in voriconazole induced periostitis.

The patient was commenced on an analgesic regime which resulted in significant symptom improvement. The possibility of discontinuing voriconazole prophylaxis was considered, but ultimately the significant benefit in terms of mitigation against fungal infection was considered to outweigh symptoms which were now well-controlled and the patient continued on lifelong voriconazole.

## Discussion

Immunosuppression post-transplantation increases patients susceptibility to infection, which may be life-threatening. Voriconazole is commonly used in both the treatment and prophylaxis of fungal infections in organ transplant recipients.

Frequently reported side effects of voriconazole include nausea and vomiting, visual disturbances, rash, abnormal liver function tests, QT prolongation, headaches and hallucinations. In 2009 the first cases of voriconazole induced periostitis were reported in 5 lung transplant patients receiving long-term prophylactic treatment with voriconazole [1]. Patients experienced diffuse bone pain, had elevated alkaline phosphatase values and imaging was consistent with diffuse periostitis. There was no history of prior rheumatological disease in this patient group and upon cessation of voriconazole symptoms resolved within days. Subsequent cases have been reported and periostitis is now a recognized but rare side effect of chronic voriconazole treatment [2,3].

Voriconazole is a tri-fluorinated compound. Several studies have shown that this increased fluoride level may play a role in the mechanism of how voriconazole causes periostitis [4]. The hydroxyl ion in hydroxyapatite is substituted for the fluoride ion, this mainly occurs in remodeling trabecular bone and in the periosteum. Fluorapatite makes resorption more difficult and fluoride stimulates osteoblasts leading to increased bone mineral density. Other antifungal drugs such as fluconazole and posaconazole are difluorinated and are not associated with periostitis supporting the mechanism of hyper fluorosis inducing periosteal pain. Serum fluoride levels are significantly higher on maintenance voriconazole treatment than in patients receiving treatment with posaconazole, itraconazole or no antifungal treatment [5].

Characteristic imaging features of periostitis include periosteal thickening, with new bone formation which tends to be dense and irregular. This is generally bilateral but may be asymmetric. Nuclear medicine imaging techniques such as bone scintigraphy or single photon emission computed tomography (SPECT) demonstrate radiotracer uptake at affected sites. The differential diagnosis for periostitis is broad, including entities such as hypertrophic osteoarthropathy, hypervitaminosis A, venous stasis and infection. The patient's clinical history (as well as the distribution of periositis) are crucial to accurate diagnosis.

Voriconazole induced periostitis typically resolves following medication discontinuation (or in some cases dose reduction) [6]. Symptomatic improvement is relatively rapid occurring in days to weeks, while the imaging appearances generally resolve over a period of months [7].

In summary, though uncommon, voriconazole-induced periostitis is an important diagnosis for radiologists to be aware of in the post-transplant or otherwise immunocompromized patient. Accurate diagnosis requires assimilation of the relevant imaging characteristics and patient history.

## Patient Consent

Informed consent has been obtained for the publication of this case report.

## Conflicts of Interest

None.
